# Genome-wide association studies in chronic venous disease: A systematic review

**DOI:** 10.1016/j.jvsv.2025.102365

**Published:** 2025-12-13

**Authors:** Chien Lin Soh, Matthew Tan, Alun H. Davies, Sarah Onida

**Affiliations:** Section of Vascular Surgery, Department of Surgery and Cancer, Imperial College London, London, UK

**Keywords:** Venous disease, Venous ulcers, Venous insufficiency, GWAS, SNPs

## Abstract

**Background:**

Chronic venous disease (CVD) arises from venous hypertension secondary to impaired venous return, causing significant morbidity and diminished quality of life. Genetic factors are likely important in the pathogenesis and susceptibility of a patient to develop CVD. This systematic review summarizes genome-wide association studies (GWASs) that investigate the link between genetic variants and CVD.

**Methods:**

A systematic review was conducted in accordance with the PRISMA guidelines, with the search dates ranging from January 1, 1994, to July 17, 2025. Abstract and full-text screening were completed by two independent reviewers, with any conflicts referred to a third senior reviewer. GWASs in adults investigating links between genetic variants and CVD were included. Exclusion criteria included patients with venous thromboembolism, arterial or diabetic disease, or animal models.

**Results:**

Thirteen studies were included after screening 517 studies from a search of PubMed, EMBASE, and Ovid. Database sources included UK Biobank, FinnGen, PopGen, and country- or hospital-specific databases with a majority Caucasian and European patient cohort. A total of 602,760 patients were identified with varicose veins and 3,664,604 control cases that were studied with GWASs and other statistical methods including a two-sample Mendelian randomization approach, functional mapping, and genetic correlations. A variety of statistically significant genetic polymorphisms were identified that can be attributed to the heritability of varicose veins affecting inflammation and immunity (eg, PPP3R1, EBF1, and GATA2), hypertension (eg, CASZ1), and vascular architecture (eg, CASZ1, PIEZO1, and STIM2). Protective variants (eg, GJD3, MMP10, and 4EBP1) were also identified in Finnish populations. However, replication studies showed that these genetic polymorphisms are not generalizable to specific populations.

**Conclusions:**

This systematic review highlights genes contributing to the development of CVD that have been identified in the literature. An improved understanding of genetic contributions to the pathogenesis of CVD may inform future diagnostics, prognostics, and personalized treatment. Further larger scale studies representative of global populations, including meta-analyses of genome-wide association datasets, are required owing to individual GWASs being statistically insufficient to draw generalizable conclusions.

**Article Highlights**

• **Type of Article:** Systematic review.
• **Key Findings:** Thirteen genome-wide association studies investigating genetic susceptibility to chronic venous disease were identified. Several genetic loci were associated with pathways related to inflammation and immunity, blood pressure regulation, and vascular structure. Protective genetic variants were reported in specific populations, but most associations lacked consistent replication across cohorts.
• **Take Home Message:** Current genome-wide association studies identify potential genetic contributors to chronic venous disease, but limited population diversity and lack of replication restrict their clinical applicability.

Chronic venous disease (CVD) is a multifactorial condition affecting a significant percentage of the global population.^1^, ^2^, ^3^ Presentations can range from mild signs such as telangiectasia to venous ulceration, summarized in the clinical component of the Clinical-Etiological-Anatomical-Pathophysiological classification.^4^ CVD is reported to have a prevalence of <1% to 40% in females and <1% to 17% in male patients.^5^ Varicose veins (VVs) are the most common clinical manifestation, affecting approximately 1% to 73% of females and 2% to 56% of males worldwide.^5^, ^6^, ^7^ Venous leg ulcers have a reported prevalence of 0.32% and a pooled incidence of 0.17% worldwide; they represent 70% of all lower limb ulcer patients in the UK, making them the most common lower limb wound type.^8^^,^^9^ CVD causes a significant socioeconomic burden, with an immense financial expenditure taking up 2% of total health care budgets of Western countries.^10^, ^11^, ^12^ CVD can progress to venous ulceration, which is associated with significant morbidity and higher mortality rates.^5^

CVD pathophysiology involves venous hypertension from impaired venous return. Alterations to venous hemodynamics result in circulatory dysfunction and aberrant inflammatory responses, leading to the physical manifestations of CVD.^1^ Venous hypertension can arise from mechanical obstruction or valvular dysfunction causing venous reflux, leading to pooling of blood in the lower extremities and manifestations of hyperpigmentation and ulceration.^13^^,^^14^

The development and progression of CVD is multifactorial, involving genetic and environmental factors. Risk factors for CVD include older age, female sex, family history of venous disease, obesity, and pregnancy, with various degrees of associations reported across various studies.^15^^,^^16^ Risks associated with a positive family history suggest genetic factors that contribute to the pathogenesis and susceptibility of a patient to developing CVD. A heritability analysis of 2701 German patients revealed an additive genetic component of inheriting CVD of 17%; however, further research into susceptibility is required to determine specific genes.^17^ Knowledge of the genetics behind the development of CVD is essential to the development of new therapies and prognostics for CVD, preventing significant morbidity and deterioration in the quality of life of these patients.^18^

Genome-wide association studies (GWASs) allow researchers to identify genetic variants and associations between common single-nucleotide polymorphisms, copy number variations, sequence variations and CVD. GWASs can be designed as population-based case-control studies—where cases with and without the disease are identified and compared.^19^ This provides an important method to study genetic differences in large populations, enriching our knowledge about critical genetic susceptibilities and biological pathways underlying venous disease.^20^^,^^21^

This systematic review aimed to summarize and describe the current literature on genetic variations in populations who suffer from CVD. Because the prevalence of CVD will only increase with an aging population, this review sought to elucidate potential genetic mechanisms that predispose to CVD development and protective variants that may one day translate into targets for therapeutic intervention.

## Methods

**Fig 1:**
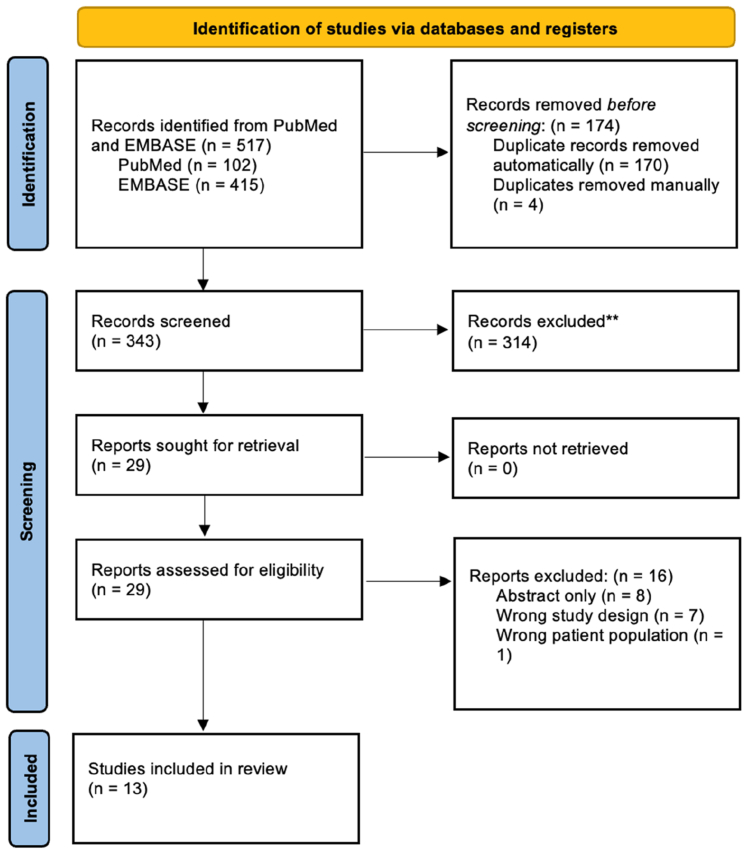
PRISMA flow chart indicating abstract and full-text screening process for final selection (n = 13).

A systematic review was conducted in accordance with the PRISMA guidelines (Fig 1) and prospectively registered on PROSPERO (CRD42024586876). A search of EMBASE and PubMed was conducted between January 1, 1994, and July 17, 2025. The study was designed to include any English language GWASs analyzing samples from patients with a CVD diagnosis confirmed on duplex ultrasound examination or coding on Biobank systems, which may be self-reported by patients. The search terms are described below (Table I). Duplicates were removed manually prior to screening.

**Table I:** Search strategy used for PubMed and EMBASE

1	("heavy leg"[All Fields] OR "heavy legs"[All Fields] OR "Venous Insufficiency"[MeSH Terms] OR "varicose veins"[MeSH Terms] OR "varicose ulcer"[MeSH Terms] OR "edema"[MeSH Terms] OR "oedema"[All Fields] OR ("edema"[MeSH Terms] OR "edema"[All Fields] OR "edemas"[All Fields] OR "oedemas"[All Fields] OR "oedema"[All Fields]) OR "varicos∗"[All Fields] OR "Venous Insufficiency"[All Fields] OR "chronic venous disease"[All Fields])
2	("Genome-wide Association Study"[MeSH Terms] OR "GWAS"[Title/Abstract] OR "gwa stud∗"[Title/Abstract] OR "genome wide association stud∗"[Title/Abstract] OR "genome wide association analys∗"[Title/Abstract] OR "whole genome association stud∗"[Title/Abstract] OR "whole genome association analys∗"[Title/Abstract] OR "genome wide scan∗"[Title/Abstract] OR "genome wide association scan∗"[Title/Abstract] OR "whole genome association scan∗"[Title/Abstract] OR "genome wide association meta analys∗"[Title/Abstract])
3	1 AND 2

1	("Varicose ulcer∗" or "leg ulcer∗" or "venous ulcer∗" or "VLU∗" or "venous wound∗" or "stasis ulcer∗" or "crural ulcer∗" or "ulcus cruris" or "ulcer cruris").mp. [mp=tx, bt, ti, ab, ct, sh, hw, tn, ot, dm, mf, dv, kf, fx, dq, cw, nm, ox, px, rx, an, ui, ds, on, sy, ux, mx, tc, id, tm]
2	(GWAS or GWA Stud∗ or Genome Wide Association Stud∗ or Genome Wide Association Analys∗ or Whole Genome Association Stud∗ or Whole Genome Association Analys∗ or Genome-wide scan∗ or Genome Wide Association Scan∗ or Whole Genome association scan∗ or genome wide association meta-analys∗).ti,ab,kf. Or genome-wide association study/
3	1 AND 2

Abstract and full-text screening were completed by two independent reviewers (C.S. and M.T.), with any conflicts referred to a third senior reviewer (S.O.). Exclusion criteria included patients with arterial, diabetic or mixed ulcers, sickle cell ulcers, or animal models. Full-text extraction that met inclusion criteria were analyzed and metadata extracted for each study (C.S. and M.T.). Authors, publication year, outcome, key findings, and single nucleotide polymorphisms (SNPs) identified with the phenotype were extracted from the articles included in the review (Table II). A meta-analysis was therefore not planned as part of this review.

**Table II:** Data extraction sheet

Study Details				Results	
Title	Country	Cohort size	Database	Overall outcome	Notable DNA expression
Genome-wide association analysis and replication in 810,625 individuals with varicose veins Ahmed, 2022^22^	United Kingdom	Cohort n = 135,514 Control n = 675,111	UK Biobank, 23andME	Pathophysiological pathways including ECM development, inflammation, angiogenesis, apoptosis Identification of similarities between VVs and SLE.	ECM genes were upregulated - COL27A1 and EFEMP1. Five inflammation-associated risk loci (ie, rs78216177)
Clinical and genetic determinants of varicose veins: Prospective, community-based study of 500 000 individuals Fukaya, 2018^23^	United States	Cohort n = 9577 Control n = 327,959	UK Biobank	Association of specific loci in genes associated with blood pressure. Identification and confirmation of risk factors for VVs (ie, height, age, sex, obesity, pregnancy, DVT).	Strong association of loci rs11121615 in CASZ1 blood pressure gene, rs2911461 in the PIEZO1 and galactosamine sulfatase enzyme (GALNS). Regulated genes by VV SNPs are rs2861819 associated with PNO1, WDR92, PLEK, and PPP3R1.
Top SNPs from the phenome-wide association study catalogue and the risk of varicose veins of lower extremities: A replication study Shadrina, 2017^24^	Russia	Cohort n = 460 Control n = 646	Russian hospital	No evidence for association with VVs of lower extremities for any polymorphisms	Nonstatistically significant associations with VVs were rs735854, rs7023329, rs2857595, rs6420094, and rs16856202 in ethnic Russians.
A genome-wide association study for varicose veins. Lee, 2022^25^	Taiwan	Cohort n = 96 Control n = 1000	Taiwan Biobank	Two statistically significant SNPs for VVs located in the DPYSL2 and VSTM2L genes identified.	Significant SNPs in VVs were the DPYSL2 and VSTM2L genes. Comparison of C2-3 and C4-6 patients revealed significant SNPs in ZNF664-FAM101 A, PHF2, ACOT11, and TOM1L1 genes.
Genome-wide association study of varicose veins identifies a protective missense variant in GJD3 enriched in the Finnish population Helkkula, 2023^26^	Finland	Cohort n = 17,027 Control n = 190,028	FinnGen	50 genetic loci identified associated with VV. Identification of missense variant with lower risk for VVs. SNP identified, which was enriched in Finnish population.	Loci identified were adjacent to known PIEZO1, SOX9, ADAM15, and CASZ116, 17, 18, 19, and 20. Novel loci were ARHGAP6, SPRX, TGFB2, and TGFBR3. Low-frequency missense variant in GJD3 associated with a lower risk for VVs. In TGFB2 and GJD3, lead SNP was enriched in Finns compared with non-Finnish non-Estonian Europeans.
Integrative analysis prioritizes the relevant genes and risk factors for chronic venous disease. He, 2022^27^	China	Cohort n = 12,021 Control n = 440,243	UK Biobank	46 lead SNPs and 26 potential causal genes for CVD identified. Associations in body mass index, height, college degree, insulin, and metformin with VVs.	Significantly downregulated expression of WDR92, RSPO3, LIMA, ABCB10, DNAJC7, C1S, and CXCL1 in CVD. Significantly upregulated expression of PHLDA1 and SERPINE1 in patients with CVD. Dysregulated expression of WDR92, RSPO3, and CASZ1 in VV.
Exploring causal correlations between inflammatory cytokines and varicose veins: A Mendelian randomization analysis. Min, 2024^28^	China	Cohort n = 29,539 Control n = 324,121	FinnGen	Genes related to systemic inflammatory modulators implicated in VV with significant associations identified.	Statistically significant association between elevated CASP-8 and VEGF-A increasing the risk of VVs. Genes 4EBP1, MMP-10 showed protective effect.
Genome-wide association analysis for chronic venous disease identifies EFEMP1 and KCNH8 as susceptibility loci. Ellinghaus, 2017^29^	Germany	Cohort n = 323 Control n = 4619	PopGen	Two novel genome-wide significant susceptibility loci for CVD identified.	Significant associations within the two genes EFEMP1 and KCNH8 (rs17278665, rs727139). Suggestive association within gene SKAP2 (rs2030136) identified.
Identifying potential drug targets for varicose veins through integration of GWAS and eQTL summary data. Cui, 2024^30^	China	Cohort n = 27,071 Control n = 642,944	FinnGen, UK Biobank	Four potential causal proteins for VVs with MR identified.	Increased expression of KRTAP5-AS1 and PLEKHA5, CBWD1 and CRIM1 genes associated with VVs. Increased expression of CRIM1 protective against VVs.
Varicose veins of lower extremities: Insights from the first large-scale genetic study. Shadrina, 2019^31^	Russia	Cohort n = 337,199 Control n = 330,241	UK Biobank, Neale lab, Gene ATLAS	12 associated loci were attributed to 13% of SNP-based heritability of VVs. Association between elevated expression of MICB and CD209 proteins, waist and hip circumference, height, weight, and both fat and fat-free mass with VVs.	- Most likely causal genes for VVs are CASZ1, PIEZO1, PPP3R1, EBF1, STIM2, HFE, GATA2, NFATC2, and SOX9.
Polymorphisms of genes involved in inflammation and blood vessel development influence the risk of varicose veins. Shadrina, 2018^32^	Russia	Cohort n = 11,570 Control n = 397,872	Russian hospital database, UK Biobank	Polymorphisms in genetic susceptibility to VVs identified.	Associations of polymorphisms rs11121615, rs6712038, rs507666, rs966562, rs7111987, rs6062618, and rs6905288 with VVs identified. Only rs11121615 reached significant association in Russian database.
Genome-wide association study in ethnic Russians suggests an association of the MHC class III genomic region with the risk of primary varicose veins. Shadrina, 2018^33^	Russia	Cohort n = 720 Control n = 693	Russian hospitals	Evidence for involvement of MHC class III genes in pathogenesis of VV demonstrated.	Variant analysis identified signal at Chr 6 in MHC class III. Associations at a genome-wide significance level were found for rs4151657 with diseases such as rheumatoid arthritis.
Exome sequencing identifies novel genetic variants associated with varicose veins. Zhang, 2024^34^	China	Cohort n = 21,643 Control n = 329,127	UK Biobank, FinnGen	13,823,269 autosomal genetic variants were obtained. 53 traits showed significant associations with identified VV-related genes (RNF5, PGBD1, TRIM31, and GNA12). Pleiotropic effects of VV-related genes linked body size, inflammation, and pulmonary function.	36 independent common variants mapped to 34 genes. 16 novel genes including TRIM10, UBE2H, TUBAL3, DUSP8, DNAH10, CAPRIN2, MSL1, ZBTB4, CIB3, SERPIND1, SHANK3, REST, CTXN3, H2AC6, PGBD1, and ABHD16A. PIEZO1 had most significant association with VVs. METTL21 A associated only with females with VVs.

*CVD,* Chronic venous disease; *DVT,* deep venous thrombosis; *ECM,* extracellular matrix; *MHC,* major histocompatibility complex; *SLE,* systemic lupus erythematosus; *VVs,* varicose veins.

**Fig 2:**
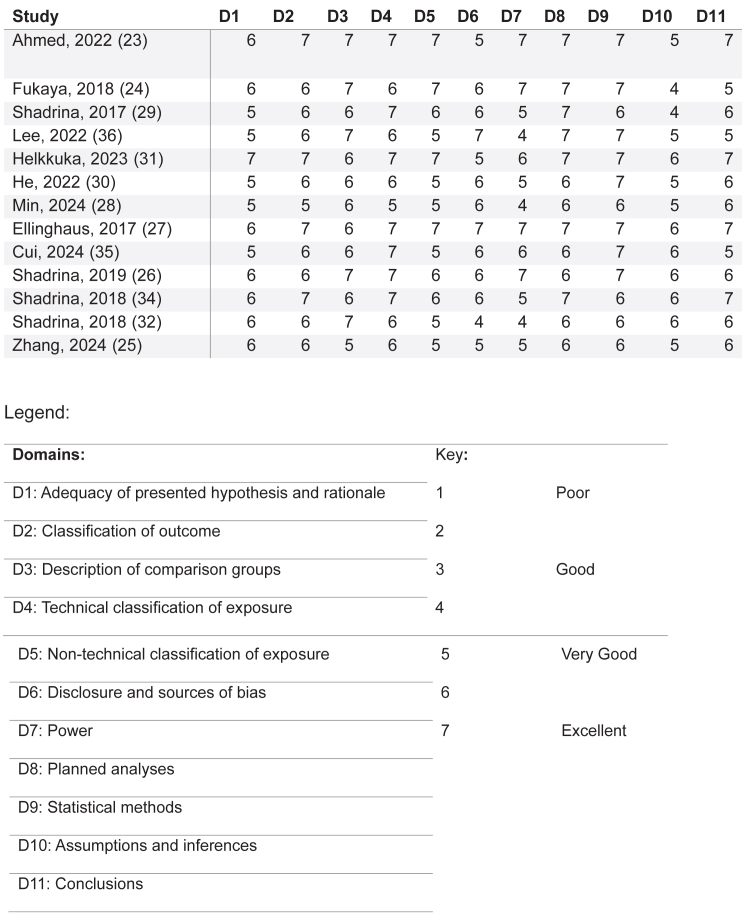
Risk of bias assessment using the Q-GENIE tool.

A risk of bias assessment was performed on included studies using the Q-GENIE tool, which includes various parameters rated according to the quality of evidence shown (Fig 2). There was no quality threshold for the selection of articles.^35^

## Results

A search of EMBASE and PubMed yielded 517 articles; 13 studies were included after full-text screening by two independent reviewers (Fig 1, Table II). Of the 13 included studies, the majority used European patient cohort databases (n = 12), with only 1 study using the Taiwan Biobank. A total of 602,760 patients' data were used in the CVD cohort compared with 3,664,604 control patients' data. A wide variety of statistical approaches, including a two-sample Mendelian randomization approach, functional mapping, and genetic correlations, were used. Many of the studies described VVs as the primary manifestation of CVD (n = 12), although there was one study describing the full spectrum of CVD, including venous ulceration.

A risk of bias assessment (Fig 2) revealed variation in the quality of the included studies assessed using the Q-GENIE tool. Of note, there were variances between each GWAS in the reporting of assumptions and inferences surrounding population selection which may have caused differences in the conclusions drawn.

### Biological pathways driving the development of CVD

**Fig 3:**
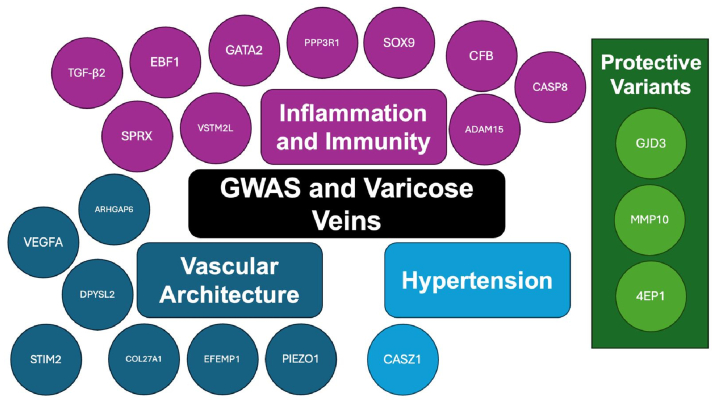
Representation of genes identified in included studies. Information on function, proteins coded for, and roles played in CVD pathogenesis for each gene can be found in the Supplementary Table. *GWAS*, genome-wide association study.

The complex genetic pathways underlying the development of the various stages of CVD were described in the included studies (Fig 3).

Genome-wide testing of a large sample set of 135,514 patients across the UK Biobank and 23andMe dataset identified 49 independent loci associated with the development of VVs. Functional variants in PIEZO1 (rs112070238 and rs8053350), which encodes ion channels detecting vascular shear stress, and enrichment of gene sets modulating extracellular matrix (ECM) (COL27A1 and EFEMP1), angiogenesis, and inflammatory pathways (DOCK8) were identified.^22^ This was further supported by a GWAS of 493,519 individuals from the UK Biobank that revealed strong associations between VVs and CASZ1, PIEZO1, and GALNS levels of expression. The SNP intergenic and intron variants rs2861819, rs2911463, and rs3101725 were shown to have further downstream effects on calcineurin pathways and vascular remodeling.^23^ A collapsing analysis identified significant associations between PIEZO1, ECE1, and FLBN7 genes with VVs, further supporting the above studies.^31^^,^^34^ The role of abnormal vasculature and angiogenesis dysfunction was further reinforced by suggestive associations with EFEMP1, KCNH8, and SKAP2 in a GWAS using independent German datasets with patients with VVs.^29^ In terms of inflammatory pathways, inflammatory biomarkers such as CASP-8 and VEGF-A were associated with an increased risk of VVs in European individuals.^28^

A Taiwan-based GWAS comparing 96 patients with CVD and 1000 sex-matched healthy controls from the Taiwan Biobank, with further subgroup analysis, identified the following significant SNPs: DPYSL2, VSTM2L, ZNF664-FAM101 A, PHF2, ACOT11, and TOM1L1. The downstream effects of these SNPs were related to dysregulation in signaling protein messengers, protein degradation, and ECM degradation.^24^ Downregulation of WDR92, RSPO3, LIMA, ABCB10, DNAJC7, C1S, and CXCL1 expression alongside upregulation of PHLDA1 and SERPINE1 genes were identified in patients with VVs. In contrast, the dysregulation of expression of WDR92, RSPO3, and CASZ1 genes were found in VV patients from quantitative polymerase chain reaction.^27^

### Discovering associations between CVD and patient factors

Two-sample Mendelian randomization analysis demonstrated association in various studies between VVs and patient factors confirmed known risk factors such as a high body mass index, sex, pregnancy, deep vein thrombosis, and hernias.^22^^,^^26^^,^^31^ Machine learning-supported association searches of the UK Biobank also identified height as a risk factor for the development of VVs.^23^ One Chinese study reported the use of insulin and metformin as a causative effect on VVs in addition to confirming other known risk factors.^27^

Sex-specific genes associated with VVs affecting female patients included METTL21 A. X-chromosome-linked genes ARHGAP6 and SRPX were also associated with VVs.^34^ Statistically significant phenome-wide associated correlations between VVs and deep venous thrombosis, thrombophlebitis, and cardiovascular disease have been demonstrated in European studies.^26^^,^^31^ The genetic overlap between VVs and autoimmune diseases such as systemic lupus erythematosus (rs17321999 [intron variant of LBH], rs4849044 [intron variant of FBLN7], rs7773004 [intergenic variant of H2BC9], and rs61863928 [regulatory region variant of ALDH7A1P4]) and rheumatoid arthritis (rs4151657 [intron variant of CFB]) have also been studied, providing insight into the underlying shared mechanisms of pathogenesis such as dysregulated immunity with other diseases.^22^

### Ethnicity-specific genetic studies in VVs

A GWAS on the Finnish population revealed 29 new genetic loci out of 50 associations including rs2836405, ARHGAP6, SRPX, TGFB2, and GJD3. Of note, a missense variant rs201955556-T in GJD3 encoding connexin proteins that facilitate wound healing was associated with lower rates of VVs.^26^

Two Russian-based studies described the genetic association of VVs in ethnic Russians. Association of a SNP rs4151657 signal within the complement factor B gene affecting the major histocompatibility complex class III subregion was described in a pilot study of 273 ethnic Russians with VVs.^33^ Phenome-wide association studies within the population of ethnic Russians with VVs had shown nonstatistically significant polymorphisms rs735854, rs7023329, rs2857595, rs6420094, and rs16856202 present in noncoding introns of the DNAs MYH9, MTAP, UQCRHP1, SLC34A1, and DISC1 respectively, suggesting potential regulatory functions underlying the development of CVD.^24^^,^^36^ Within the Russian population, polymorphism rs11121615, an intron variant of CAZS1, was identified as a nominally significant expression, affecting genes involved in vasculature and inflammatory response.^32^

### Potential therapeutic targets for VVs

Protective variants against VVs identified include CRIM1, 4EBP1, MMP-10, and a missense variant in GJD3.^26^^,^^28^^,^^30^ Identification of pharmacologically relevant genes among identified SNPs included KRTAP5-AS1, PLEKHA5, CBWD1, and CRIM1 in the development of VVs. These SNPs represent a valuable target for potential future drug development.^30^

Drug development targets have already been investigated through drug target enrichment analysis. These analyses have revealed potential biological pathways that mediate VV development that can be addressed as genetic targets, including pathways relating to immune response, angiogenesis and apoptosis. Known pharmaceutical interactions with genes including CDK10, COL27A1, GABBR1, KCNJ2, MAPK10, OPRL1, TNC, and VEGFA were also identified.^22^

## Discussion

This systematic review encompassing 13 studies examining individuals with CVD mainly focuses on VVs. There is a great need for a better understanding of the genetic mechanisms underpinning VVs to develop new therapeutic and preventative strategies. With the advent and development in sequencing technology, GWASs have elucidated common genetic risk factors for VVs with >30 risk loci in large-scale GWASs.^23^

The pathogenesis of CVD is conventionally thought to arise from disruption of vascular endothelium owing to venous hypertension, altered immune response, chronic inflammation, remodeling, and angiogenesis. The included GWASs identify key genes in modulating steps of this pathway, especially with vascular remodeling, chronic inflammation and the modulation of ECM regulation.^25^^,^^37^ This systematic review confirms known associations in the literature whilst highlighting new genetic variants such as DPYSL2, VSTM2L, EFEMP1, KCNH8, and SKAP2 in the susceptibility to CVD.^29^^,^^38^

VVs are associated with disruption of the ECM, with deposition of ECM proteins such as collagen and elastin increased compared with normal vein walls.^39^ Of note, the involvement of SNPs related to EFEMP1 and PIEZO1 has been well-described in the literature by several papers. A key gene COL27A1, which is a fibrillar collagen in blood vessels, is reduced in VV samples and significantly overlaps with pathways such as EFEMP1, which encodes the ECM glycoprotein fibulin-3, resulting in dysregulation of venous walls.^22^ Variants of EFEMP1 are found in pathways linked to ECM organization and molecules associated with elasticity of structures, therefore associating the gene with the architecture of venous walls.^40^ In terms of angiogenesis, the gene PIEZO1, which encodes a vascular mechanosensory channel, is implicated in vascular development and maturation, associated with VVs and lymphedema.^22^^,^^23^ These downstream effects on the transcriptome are recognized where PIEZO1 is implicated in the development of VVs; there is increased messenger RNA expression of PIEZO1 in clinical VVs, exome sequence database studies reported increased risk of VVs for individuals with protein-truncating variants in PIEZO1 and gene deletion studies in animal models alleviated VVs.^41^^,^^42^ This protective effect against VVs was thought to arise from a decrease in vascular permeability and leukocyte infiltration in models without PIEZO1, linking the gene with angiogenesis and vascular permeability. Genes such as CASZ1 are also implicated in VVs, with a positive association with CVD.^43^ CASZ1 plays important roles in functions relating to tissue differentiation and aldosterone antagonism. These studies did not demonstrate any increase in CASZ1 messenger RNA in patients with VVs or any association with VVs and CASZ1 PTVs.^41^^,^^42^

A systematic review revealed various associations between other medical conditions and VVs. Individuals with deep venous thrombosis and VVs have been shown to share familial risk in a nationwide Swedish study.^44^ For example, the identification of F5 p.Arg534Gln (factor V Leiden) gene has been identified as an overlapping locus of focus for both VVs and deep venous thrombosis.^45^ There have also been reports of associations at a polygenic level with a significant correlation between VVs and arterial disease such as peripheral arterial disease and abdominal aortic aneurysms, in addition to diseases such as systemic lupus erythematosus.^22^^,^^38^ These associations represent a valuable genetic link between the pathogenesis of VVs and autoinflammatory diseases, which demonstrate underlying shared pathways in the development of VVs and potential treatment targets.

Because there are fundamental differences in the genetic composition of patients of different ethnicities, the majority of studies reported examine European cohorts of patients. The only Asian-based study of a Taiwanese cohort of patients comparing 96 patients with CVD from a local hospital with controls from the Taiwan Biobank identified a distinct set of significant SNPs associated with VVs, which differed entirely from the European studies.^24^ Representative global studies are required to fully understand the genetic basis of venous diseases given the genetic variation between populations which can contribute to this pathology.

Although there is a contentious but known familial risk for VVs, there are insufficient genetic studies to explain this heritability with sufficiently robust evidence.^46^, ^47^, ^48^ In addition, these studies are often studying individuals of European ancestry, which represent a limited population and are not generalizable to a global population.

### Limitations

There are inherent limitations to GWASs, such as limitations of any conclusions to the specific populations studied and insufficient statistical significance owing to limited power and sample sizes. There is significant variance in results between each country as demonstrated, owing to inherent differences in the genetic basis of each ethnicity. GWASs generate hypothetical associations between SNPs and phenotypes, but further downstream -omics studies must be linked to demonstrate reliable causality. There may also be complex environmental influences on GWASs which may result in false associations and epidemiological bias. These concerns regarding false-positive associations are valid, and further fine-mapping and functional studies may identify contributions of these genes to disease susceptibility. As described, variations in methodology can result in different results from the same resources, emphasizing the importance of definitions of the researched condition.

Of note, two separate biobank studies using the UK Biobank investigating genetic correlations with VVs describe two different sets of genetic correlations. Further review of the methodologies of each paper reveal that one used a self-reported genetic database using patient-reported diagnosis as a validation study for the UK Biobank data, which may increase over-reporting of the incidence of VVs. The differences in the use of ICD codes in the studies may have also contributed to the differences in genetic outcomes.^22^^,^^23^ These variances make it difficult to draw firm conclusions regarding the true underlying genetic mechanisms of VVs, because the results were dependent on defined parameters in the methodologies.

Further development, in-depth research, and aggregated studies are required to confirm and replicate the findings from these studies, especially in non-European populations. This work will provide support for development of translational research and large-scale clinical trials to identify therapeutic targets for treatment and prevention of VVs.

## Conclusions

This systematic review demonstrates that key genes contributing to the development of CVD can be identified from GWASs; however, the exact nature of their involvement requires upstream and downstream -omics analysis to understand their role in the manifestations of CVD. Individual GWASs are not statistically sufficient or generalizable to global populations owing to the lack of diversity, and further larger scale studies, including meta-analyses of genome-wide association datasets, are required to draw comprehensive associations.

## Author contributions

Conception and design: CS, MT, AD, SO

Analysis and interpretation: CS, MT

Data collection: CS

Writing the article: CS, MT

Critical revision of the article: CS, MT, AD, SO

Final approval of the article: CS, MT, AD, SO

Statistical analysis: Not applicable

Obtained funding: Not applicable

Overall responsibility: SO

## Funding

None.

## Disclosures

None.
