# Supplementary material for: Genome-wide association studies in chronic venous disease: A systematic review
Source: J Vasc Surg Venous Lymphat Disord. 2025 Dec 13;14(2):102365. doi: 10.1016/j.jvsv.2025.102365 (PMC12830204; doi:10.1016/j.jvsv.2025.102365)
Supplement: Supplementary Material [file mmc1.docx]

Supplementary File:

Table clarifying additional functions of genetic factors included in Figure 3. Descriptions of functions, which proteins coded, roles played with pathogenesis of CVD.

| **Genetic Factors** | **Function** | **Proteins Coded** | **Roles Played in Pathogenesis of CVD** |
| --- | --- | --- | --- |
| CASZ1 (1) | Cardiac development, vascular assembly, lumen morphogenesis | Zinc finger transcription protein | Strong association with CVD through angiogenesis and vascular assembly. |
| PIEZO1 (2) | Touch and pain sensation, blood pressure regulation and cell homeostasis | Cation channel, mechanosensitive channel responsive to Ca2+ influx | Associated with regulation in vascular tone and lymphatic circulation. |
| EFEMP1 (3) | Epidermal growth factor activity, connective tissue integrity and cell growth regulator. | Extracellular matrix glycoproteins | Associated with venous wall development |
| COL27A1 (4) | Cartilage calcification and transition of cartilage to bone | Fibrillar collagen family | Associated with extracellular matrix development |
| STIM2 (5) | Intracellular signalling and gene expression | Endoplasmic reticulum protein | Associated with aging and autoimmune conditions. |
| DPYSL2 (6) | Facilitates neuron function, microtubule assembly | Collapsin response mediator protein 2 | Possible association with angiogenesis |
| VEGFA (7) | Proliferation and migration of endothelial cells | Vascular endothelial growth factor A | Associated with angiogenesis |
| ARHGAP6 (8) | Regulation of actin modelling | Rho GTPase activating protein 6 | N/A |
| TGF-B2 (9) | Controls tissue homeostasis and cell proliferation – diverse range of functions | Multifunctional cytokine | Associated with venous thromboembolism development and fibrosis |
| SPRX (10) | Cell wall metabolism and function | Missense variant | N/A |
| EBF1 (11) | B-cell differentiation and signal transduction | Transcription factor | Associated with immune response |
| VSTM2L (12) | Regulation of neuron apoptosis | Protein that binds to humanin | May not be directly involved in varicose vein pathogenesis |
| GATA2 (13) | Lymphatic development | Transcription factor | Associated with regulation of angiogenesis and lymphangiogenesis |
| PPP3R1 (14) | Involved in phosphatase binding activity and calcineurin-NFAT signalling cascade | Calcineurin subunit B type 1 | Associated with immune response or inflammation |
| SOX9 (15) | Protein for embryonic development | Transcription factor | Currently unclear association |
| CFB (16) | Regulation of cell senescence | Complement factor B | Currently unclear association |
| ADAM15 (17) | Involved in cell adhesion and cytokines | Disintegrin and metalloproteinase domain-containing protein 15 | Associated with vascular barrier dysfunction |
| CASP8 (18) | Involved in cell apoptosis | Caspase-8 | Associated with immune response |
| GJD3 (10) | Involved with gap junctions | Gap junction protein delta 3 | Missense variant associated with lower risk of varicose veins |
| MMP10 (19) | Involved with tissue remodelling, wound repair and atherosclerosis. | Matrix metalloproteinase | Associated with connective tissue remodelling |
| 4EP1(20) | Tumour suppressor activity | Eukaryotic translation initiation factor 4E-binding protein 1 | No clear association |

References:

1. Liu T, Li T, Ke S. Role of the CASZ1 transcription factor in tissue development and disease. European Journal of Medical Research. 2023;28(1):562.

2. Thien ND, Hai-Nam N, Anh DT, Baecker D. Piezo1 and its inhibitors: Overview and perspectives. European Journal of Medicinal Chemistry. 2024;273:116502.

3. Livingstone I, Uversky VN, Furniss D, Wiberg A. The Pathophysiological Significance of Fibulin-3. Biomolecules. 2020;10(9).

4. Costa D, Andreucci M, Ielapi N, Serraino GF, Mastroroberto P, Bracale UM, et al. Molecular Determinants of Chronic Venous Disease: A Comprehensive Review. Int J Mol Sci. 2023;24(3).

5. Berna-Erro A, Jardin I, Salido GM, Rosado JA. Role of STIM2 in cell function and physiopathology. J Physiol. 2017;595(10):3111-28.

6. Pham X, Song G, Lao S, Goff L, Zhu H, Valle D, et al. The DPYSL2 gene connects mTOR and schizophrenia. Translational Psychiatry. 2016;6(11):e933-e.

7. Lungu CN, Mehedinti MC. Molecular Motifs in Vascular Morphogenesis: Vascular Endothelial Growth Factor A (VEGFA) as the Leading Promoter of Angiogenesis. Int J Mol Sci. 2023;24(15).

8. Prakash SK, Paylor R, Jenna S, Lamarche-Vane N, Armstrong DL, Xu B, et al. Functional analysis of ARHGAP6, a novel GTPase-activating protein for RhoA. Hum Mol Genet. 2000;9(4):477-88.

9. Deng Z, Fan T, Xiao C, Tian H, Zheng Y, Li C, et al. TGF-β signaling in health, disease and therapeutics. Signal Transduction and Targeted Therapy. 2024;9(1):61.

10. Helkkula P, Hassan S, Saarentaus E, Vartiainen E, Ruotsalainen S, Leinonen JT, et al. Genome-wide association study of varicose veins identifies a protective missense variant in GJD3 enriched in the Finnish population. Commun Biol. 2023;6(1):71.

11. Nechanitzky R, Akbas D, Scherer S, Györy I, Hoyler T, Ramamoorthy S, et al. Transcription factor EBF1 is essential for the maintenance of B cell identity and prevention of alternative fates in committed cells. Nature Immunology. 2013;14(8):867-75.

12. Lee ML, Liang C, Chuang CH, Lee PS, Chen TH, Sun S, et al. A genome-wide association study for varicose veins. Phlebology. 2022;37(4):267-78.

13. Kazenwadel J, Betterman KL, Chong CE, Stokes PH, Lee YK, Secker GA, et al. GATA2 is required for lymphatic vessel valve development and maintenance. J Clin Invest. 2015;125(8):2979-94.

14. Shadrina AS, Sharapov SZ, Shashkova TI, Tsepilov YA. Varicose veins of lower extremities: Insights from the first large-scale genetic study. PLoS Genet. 2019;15(4):e1008110.

15. Helkkula P, Hassan S, Saarentaus E, Vartiainen E, Ruotsalainen S, Leinonen JT, et al. Genome-wide association study of varicose veins identifies a protective missense variant in GJD3 enriched in the Finnish population. Communications Biology. 2023;6(1):71.

16. He C, Wang X, Jiang B, Zhu M, Zhang H, Duan Y, et al. Complement Factor B (CFB) inhibits the malignant progression of lung adenocarcinoma by downregulating the Ras/MAPK signaling pathway. Arch Biochem Biophys. 2024;760:110130.

17. Lucas N, Najy AJ, Day ML. The therapeutic potential of ADAM15. Curr Pharm Des. 2009;15(20):2311-8.

18. Pang J, Vince JE. The role of caspase-8 in inflammatory signalling and pyroptotic cell death. Semin Immunol. 2023;70:101832.

19. Komosinska-Vassev K, Olczyk P, Winsz-Szczotka K, Kuznik-Trocha K, Klimek K, Olczyk K. Age- and gender-dependent changes in connective tissue remodeling: physiological differences in circulating MMP-3, MMP-10, TIMP-1 and TIMP-2 level. Gerontology. 2011;57(1):44-52.

20. Musa J, Orth MF, Dallmayer M, Baldauf M, Pardo C, Rotblat B, et al. Eukaryotic initiation factor 4E-binding protein 1 (4E-BP1): a master regulator of mRNA translation involved in tumorigenesis. Oncogene. 2016;35(36):4675-88.
